# Methods for serial analysis of long time series in the study of biological rhythms

**DOI:** 10.1186/1740-3391-11-7

**Published:** 2013-07-18

**Authors:** Antoni Díez-Noguera

**Affiliations:** 1 Departament de Fisiologia, Facultat de Farmàcia, Universitat de Barcelona, Avenue Joan XXIII 27-31, 08028, Barcelona, Spain

**Keywords:** Methods of analysis, Long time series, Serial analysis

## Abstract

When one is faced with the analysis of long time series, one often finds that the characteristics of circadian rhythms vary with time throughout the series. To cope with this situation, the whole series can be fragmented into successive sections which are analyzed one after the other, which constitutes a serial analysis. This article discusses serial analysis techniques, beginning with the characteristics that the sections must have and how they can affect the results. After consideration of the effects of some simple filters, different types of serial analysis are discussed systematically according to the variable analyzed or the estimated parameters: scalar magnitudes, angular magnitudes (time or phase), magnitudes related to frequencies (or periods), periodograms, and derived and / or special magnitudes and variables. The use of wavelet analysis and convolutions in long time series is also discussed. In all cases the fundamentals of each method are exposed, jointly with practical considerations and graphic examples. The final section provides information about software available to perform this type of analysis.

## Background

Virtually all living beings exhibit circadian rhythms that are manifested as oscillations with periods close to 24 hours in almost all their physiological variables. The study of these rhythms is thus based on the characterization and quantification of these oscillations, and, for this, one can use various statistical and mathematical techniques [1], which often come from wave analysis theory. These techniques include quantification of various descriptive parameters in the time domain: average values, variability (variance) of the oscillation, amplitude, the duration of the activity phase (alpha), the mean value in the alpha phase, and a whole host of indices derived from these and other similar magnitudes. Another set of techniques to study the characteristics of the rhythm in the domain of frequency are: spectral analysis, periodic regression (fitting to sinusoids), periodogram, calculation of phases, etc. In all these cases the presence of a periodic process is assumed, which is repeated with a certain frequency, either known or unknown.

In many such studies, a descriptive analysis is enough to characterize the circadian rhythm of one or more individuals under certain circumstances, and it is common to compare these parameters among different individuals that may be under different environmental conditions or under different experimental conditions. But a different situation occurs when performing long experimental studies in which a variable is recorded (usually spontaneous motor activity) for long periods of time [2]. In these cases the main objective of the study is usually to observe and quantify the evolution and the changes in the rhythm throughout the study. To do this, one applies the aforementioned techniques, repetitively at different times of the period analyzed. So we can “cut” the recorded data series in several sections to be analyzed individually and then compare the results.

However, when very long and practically continuous registers are available, it is of greater interest the study of the changes that occur over the time, in a continuous or quasi-continuous (daily) way. This is particularly frequent when monitoring changes of phase, in response to external stimuli, or to environmental changes. But virtually all variables from the descriptive analysis of a circadian rhythm can be continuously monitored over time [3]. This type of analysis provides invaluable information to the knowledge of the circadian system and their responses. This is also of great interest in the study of the processes of maturation or degeneration that occur throughout the life of the individuals of a given species.

In this article we will review the techniques that allow the analysis of circadian rhythms through a relatively long period of time, considering the most frequently used and also their software availability and its informative value. In all cases, the techniques will be introduced briefly along with some comments about its features and applicability. To properly illustrate their characteristics, both synthetic and real experimental datasets will be used in examples, to highlight some features of their behavior.

### The serial analysis concept

The study of changes in the characteristics of a rhythm over time is a kind of “multiresolution analysis” since the factor “time” is considered at two clearly differentiated levels: one level of resolution is related to the circadian rhythm itself (approximately 24 hours) and the other is the level of resolution of the timescale in which it is intended to detect changes of circadian rhythm, which can range from a few weeks to several months or years. In some special cases these changes are also periodicities like, weekly, monthly or seasonal. This leads to a set of techniques in which the primary data series is constituted by the results from the daily analysis of the original data, and will not be considered in this article.

The first step necessary to perform the analysis over time is to segment the data series into different sections with the same size, in each of which an analysis in the “circadian range” will be done. In this way we will obtain a series of results that will constitute a new series in a “global range of time”. This type of analysis is often applied to data sets that are analyzed in the circadian range by fitting a sine function in what is called “serial analysis” or “serial section analysis” [4-6]. Although strictly speaking the term “serial analysis” is widely used for the investigation of specific sequences in ordered collections of data (e.g. analysis of base sequences in genetics) we consider that we can keep using this term, without ambiguity, in the area we are now considering. Another possible term that we can use for this analysis could be that of “sequential analysis” since the analysis is performed sequentially in one section after another, but this term also has a specific meaning in statistics different from what we are considering here. Here we will generalize the use of the term “serial analysis” to cases where the analysis of successive sections will not be based on fitting the data to a sinusoid in the circadian range. This will be the case of the periodograms, phase analysis, etc.

In order to establish a consistent formal notation, we will consider a generic variable as X (temperature, motor activity, plasma concentration, blood pressure, etc.), whose different values over time are expressed as a function of time x(t). The series (being analyzed) is constituted by the successive observations made at different times t_1_, t_2_ … t_i_ (i = 1 … N) giving the values x (t_1_), x (t_2_), … x (t_i_). For simplicity, in the case of a discrete series of values, we can do x_i_ ≡ x (t_i_), so that the series will consist of {x_1_, x_2_ … x_i_}. In all cases (and if not otherwise stated) it is assumed that the series is uniformly sampled with a constant sampling interval Δt = t_i_-t_i-1_. In this series, we can define different sections as subsets of m values of X, which are generically designated with the letter Y. Thus the series Y_k_ (i.e. the section k) will be formed by l values of X, starting at the sample k, so that: Y_k_ = {y_1_, y_2_ … y_l_}_k_ = {x_k_, x_k+1_, … x_k+l-1_}. In the next part will show a more precise formulation, depending on the characteristics of the section.

Regarding the nature of the variable X, practically all methods of analysis are defined for continuous quantitative variables, like temperature or blood pressure, but in many cases the variable has a discrete quantitative (countable) character, as the number of movements. In these cases one can consider that the value of the variable (e.g. the number of counts in a fixed time) is proportional to the probability that an individual makes a move or, in any case, is proportional to the abstract variable “the tendency to move”. According to this, one can treat these variables as a continuous one. To be strictly correct these values should be transformed by the application of a linear filter [6], but this should be necessary only for extremely discrete values (only 2 or 3 levels). For values greater than 5 they can be used directly without any transform.

### Characteristics and definition of sections

**Figure 1:**
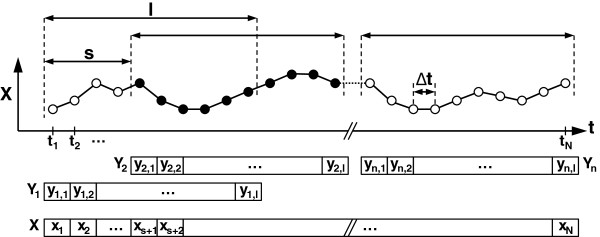
**Schematic representation of sections.** X is the original data set. Filled circles correspond to the elements of the section Y_2_. In this example the step s = 4, and the length l = 10, so they are overlapping sections (s < l). Δt is the sampling interval (see text for details).

In the previous paragraphs a simple way to define the sections has been considered, taking into account only its position in the overall series X and its length l. Actually the definition of the different sections is a bit more complex since it is necessary to consider the magnitude (s) of the displacement (jump or step) between successive sections. Both the length of the section (l) and the magnitude of the shift (s) have a close relationship with the value of the period T, to be used for the analysis. So the generic definition of successive sections Y_1_, Y_2_ … Y_j_ (j = 1, 2 … n; n = N · l/s + 1) is Y_j_ = {y_1_, y_2_, … y_l_} _j_ = {x _(j-1)s+1_, x _(j-1)s+2_ … x _(j-1)s+l_}, which is shown more clearly in Figure 1. Thus y_1_, y_2_, … y_l_, represent the elements of any section, while appending a subscript j, then y_j,1_, y_j,2_, … y_j,l_ represent the elements of a particular section Y_j_. Hereafter and unless otherwise indicated, all values of the intervals (s, l, and T) are expressed in number of samples, so that their value in real time units will be calculated by multiplying by the value of Δt sampling interval. From each section a characteristic value of the circadian rhythm will be obtained. The array of these values {z_1_, z_2_, … z_n_} will be the new Z series whose evolution is analyzed over time and that is the objective of the serial analysis.

**Figure 2:**
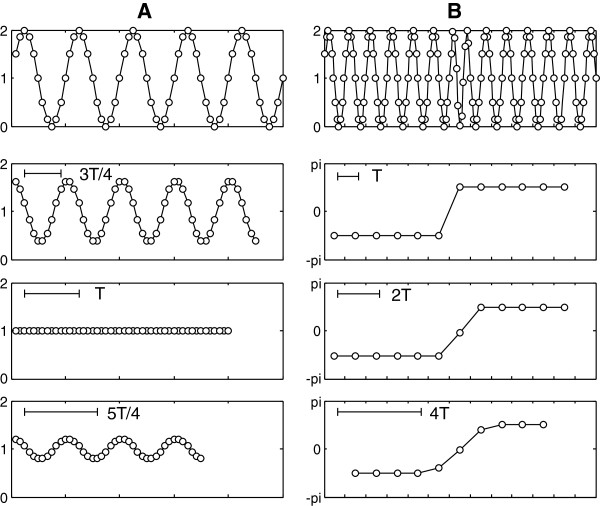
**Effect of the length of the section on the result series values Z.** In the top row, the original series (X), and in the lower rows the resulting Z variables, using sections with different lengths. In **A**, X is a sinusoidal function with period T = 12. The graphs below, show the mean value calculated with a step s = 1, and l values of 3 T/4, T and 5 T/4. Although the real mean remains constant, for different values of l, an oscillation is present. In **B**, X is a sinusoidal function with period T = 12 with a phase delay in the middle of the series. In the lower graphs the phases calculated with sections of length T, 2 T and 4 T, are shown. A smoothing of the real phase shift is visible when increasing the length of the section.

The selection of the length l of the section and the value of the displacement s must be done carefully, taking into account the following considerations. The length value must be an integer multiple of the period used for analysis in the circadian range (T, used to be the period corresponding to 24 h), otherwise false oscillations appear in the Z series, which will not be more than a residual of the original oscillation. Figure 2A shows the effect of the length of the section on the series of results Z. In the example the variable Z corresponds to the mean value of the sections, i.e. z = m_Y_. The data set X is a sinusoidal function with period T = 12 and a constant of 1. The step value is set at s = 1, so that the sections overlap in order to obtain the “instant” value of the mean for each time point. The lengths l that are tested correspond to l = 3 T/4, T and 5 T/4. It can be clearly seen that for l = T, the Z value reflects the actual value of the average over the entire range studied, whereas for l = 3 T/4 and 5 T/4, an oscillation occurs in the value of m with the same period as the original series X.

For the selection of the jump s, one must take into account whether the value calculated at circadian level, could be considered as a continuous process that exists all the time, or exists only for intervals of length equal to T. In the first case it can be considered that there exists an “instant” value (e.g. the heart rate), which allows the calculation of Z for each point of the original series, i.e. Z can take values at any time between 0 and T. In the second case (e.g. the calculation of an amplitude), it would be advisable to use a value of s = T, to avoid the errors discussed in the previous paragraph.

When one uses values of s lower than l, an overlap occurs between the successive sections, which should be taken into account when one checks the statistical significance of the values of the resulting variable Z. This applies only where the variable Z, calculated from sections, is defined under some hypothesis, such as being different from zero, or a fixed value, which allows the estimation of the statistical significance. This could be the case that Z corresponds, for example, to an average or a variance. If there are no overlapping sections, the significance level p, for each value of Z, can be calculated independently, because the data sets used to calculate each value of Z are different from each other. Conversely, in the event of having overlapping sections (s < l), the successive values of Z, share several values of X. Thus a part of a data set used to calculate a Z value can be used to calculate other Z values. In these cases, to maintain a fixed level of significance, a correction should be applied to calculate the level of significance for each single test of Z, depending on the number of statistical tests (Z values) that share a value of X for its calculation. If we call this number m (m = integer(s/l)-1), the corrected significance level will be calculated by Sidak’s formula [7] p_m_ = 1-(1-p)^1/m^ which, for values of m > 3, is approached by the Bonferroni correction [8] p_m_ = p/m, where p is the probability level for the overall set of tests.

The last aspect to be considered is the expected rate of change in the process being studied. In this case a balance is required between the minimum length of the section needed for calculating the parameter Z (e.g. the phase), the magnitude of the jump s, and the expected rate of change. The rate of change is defined by the time-constant of the process, which in physics is defined as the time required for the variable to reach a fraction of 0.632 of its final value (0.632 = 1-1/e). In order to follow the changing process properly, the value of the step s must not exceed the expected time-constant of the process, and it is recommended to keep it below half that value. With regard to the choice of the length of the section l, this should be kept as short as possible in order to detect changes in the moment at which they occur. Anyway, this value will be conditioned by the length required for the calculation of the parameters corresponding to Z. Thus, for example, in the case of a serial periodogram, it is necessary to use a length l ≥ 10 T, to perform the calculation confidently.

It has to be kept in mind that, when very long sections are used, a smoothing will occur in the series of values of Z. This smoothing is equivalent to the average of a number of Z values equal to the number of complete cycles T included in each section (see Figure 2B). Thus a value of Z obtained from sections of length l = mT, is equivalent to the smoothing of m elements calculated in the Z series (calculated with a length l = T). This effect may be desirable in cases where the variable Z is very unstable, but it should be avoided when dealing with rapidly changing processes. In many cases where the studied variable may change from cycle to cycle, one single cycle is enough for the determination of Z values, and very often the three intervals take the same value, i.e. T = l = s.

A final consideration relates to the mode of presenting the results graphically that, in general, differs from the regular cartesian graph with the time axis in the abscissas. Since this type of analysis is performed on large data sets, which are usually represented as actograms or “double plotted graphs”, most often the results series Z is represented, in parallel to the above graphs. That is, the variable Z is represented in abscissas, and vertically, starting from the top, successive values of Z (derived from sections) are shown in descending order. When the variable Z corresponds to a time (or phase) within the cycle T, the horizontal axis goes from 0 to T, and often it is superimposed to the “Double plotted graph” of X to facilitate the location of phases within the series analyzed.

### Analysis of sections and types of variables

As already mentioned the “results series” Z is formed by the sequence of values resulting from the analysis of successive sections and obtained from the segmentation of the original series X. Thus, Z is a variable, or a feature of each section, as could be the average amplitude of a sinusoidal oscillation, the period, the variance, etc. In order to describe the different possibilities, five types of results can be considered: scalar magnitudes, angular magnitudes (time or phase), magnitudes related to frequencies (or periods), periodograms, and derived and/or special magnitudes and variables. Each one of these types has some common formal and methodological properties that will be discussed below.

There is another type of analysis called “wavelet analysis” which also allows seeing the evolution of the characteristics of the circadian rhythm through time. Although it uses a very specific methodology that is different from above, a comment on this technique will also be included jointly with the convolutions.

#### Scalar magnitudes

**Figure 3:**
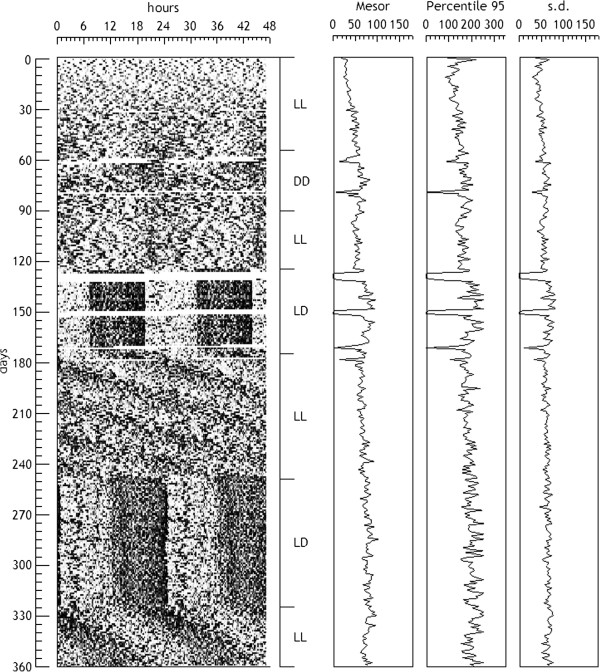
**Serial analysis of a 360 days record of motor activity of a rat submitted to different light patterns.** Sections are s = l = T = 1440 min. The calculated variables are shown at right: mesor, percentile 95% and standard deviation.

They are simple magnitudes representing a feature of the rhythm analyzed at a circadian level. Usually non-overlapping sections are used, with s = l = T (daily sections). They have clearly descriptive purposes, and among the most used are: the mean, the median or a percentile value, but also the minimum value, the maximum value, the total (daily) sum, the variance or the range can be used. Figure 3 shows the evolution of some of these parameters for a real data series.

In case of using sections with a length different of T, it is very important that the length l be multiple of T (as already discussed in the previous section) to avoid the presence of false fluctuations in the analyzed parameter. Another consideration to be taken into account when trying to plot individual values of the series (e.g.: minimum or maximum values) will be the application of filters, since in the case of using values calculated from the entire section, random variations or noise present in the data is compensated by the calculation itself, whereas when the result is a single value from the series it can be seriously disturbed by the noise. Applying a filter to the whole data set before the calculation is a practice advisable in most cases, as it reduces the noise in the series, decreasing the residual variance and increasing the accuracy in the estimation of parameters.

The use of numeric filters constitutes a broad field in the theory of signal analysis and there is a wide variety of types and features, and highly specific filters can be designed for the removal (or amplification) of definite frequency ranges. Except in special cases, the use of very specialized filters can modify the presence of frequency components in an unwanted manner, distorting seriously the characteristics of the series being analyzed. Instead, what is really advisable is the use of simple filters for noise reduction, which is usually constituted by high frequency components.

**Figure 4:**
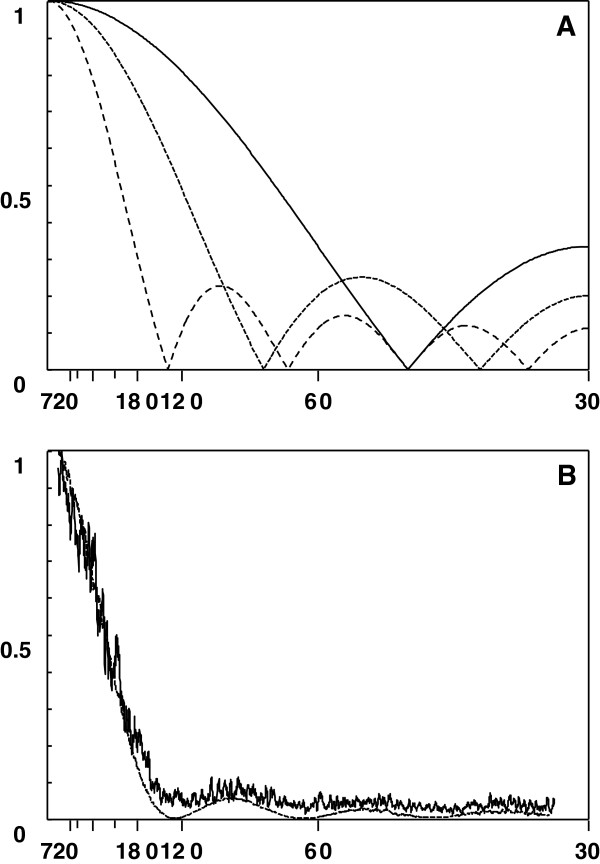
**Transfer functions corresponding to the application of a moving average in a series with a sampling interval of 15 min.** Periods are represented in the abscissas (in minutes), and in the ordinates is the ratio of pass for different periods. In **A**, continuous line is for an interval of 5 points (1 h), dotted line for 9 points (2 h) and dashed line for 17 points (4 h). In **B**, real transfer function for pink noise using a moving average (dotted line) and a moving median with the same interval (continuous line).

Thus, it will consist in applying low-pass filters, the simplest one of them being the moving average. With a moving average, the new filtered data (x') will be obtained by averaging the n values before and the n values after the reference point, according with the formula x'_i_ = (x_i-n_ + … + x_i_ + … + x_i+n_) / (2n +1). The moving average produces a smoothing effect on the data that removes high frequency components, depending on the amplitude of the interval M = 2n + 1, as shown in Figure 4A. The choice of n will depend on the highest frequency to be detected in the subsequent analysis. To determine the effect of smoothing we can calculate the transfer function H(f), according to the formula: H(f) = abs [sin(π · f · M) / (M · sin(π · f))], where H(f) represents the relationship between the signal before and after filtering for each frequency f, where a f = 1 is the frequency corresponding to two sampling intervals: f_N_ = 1/(2 Δt), that is known as the Nyquist frequency. As an example, for a sampling interval of Δt = 15 min., a frequency f = 0.4 corresponds to a period T = 1/f = 1/(0.4 · f_N_) = 2 Δt /0.4 = 2 · 15/0.4 = 75 min. As seen in Figure 4A, the frequency reduction is not uniform and has a strong ripple, but remains at very acceptable levels. It is possible to design a filter with a much flatter response, but it is actually much more complex. Regarding the sample interval, we must remember that the shorter period that one can analyze in a series is determined by the Nyquist frequency, giving a value of T =1/f_N_ = 2 Δt.

In real series there is a reduction of low frequencies somewhat higher that in theory, as shown in the example of Figure 4B, calculated from a series of “real pink noise” (1/f). The same figure shows the effect of applying another interesting filter: a running median. This type of filter is highly recommended because it eliminates very effectively spurious or aberrant data from the series. In the case of the moving average, these values can produce a displacement that may distort the series significantly. In addition, the moving median produces a similar reduction at high frequencies as the moving average does but with a noisy transfer function. In spite of this, the filtering effect is practically unaffected. For these reasons it would be always advisable to smooth out the data with a running median before proceeding to the serial analysis of data.

#### Angular magnitudes (time or phase)

Tracking a characteristic moment of the circadian cycle along successive days is usually one of the most characteristic aspects of numerous circadian rhythm studies. The choice of this point is controversial, and there is no consensus in defining a unique moment that can be used in all cases. In many cases where the motor activity of an animal is recorded, the beginning of the active phase is usually a very stable indicator of the phase of the animal [9,10]. In other cases, for certain diurnal animals, the offset of activity is better [11], while in other cases more complex patterns hinder the selection of a characteristic point. In many cases the right selection of one point or the other determines the quality of results, as in the case of the calculation of phase changes to obtain a phase response curve [12].

When the patterns are less contrasted or softer, or the variables change with a slower speed (e.g. body temperature), it is usual to adjust an envelope to the data [13] or, better, find a parameter indicating the central phase of the cycle. This is usually done by the estimation of the acrophase [14,15], which results from fitting the data of the section to a sinusoidal function. The acrophase represents the instant at which the function reaches its maximum value. In the case of a uniform or regular sampling, the acrophase is easily calculated from the formula:

$$\phi =\arctan\frac{\sum_{i=1}^{n}y_{i}\sin\frac{2\mathrm{\pi i}}{n}}{\sum_{i=1}^{n}y_{i}\cos\frac{2\mathrm{\pi i}}{n}}$$

In many cases when the pattern of the studied variable is far from sinusoid, the time of acrophase will not necessarily coincide with the maximum value of the actual series, and for this reason many authors criticize its use, but we will see later that the phase φ (or acrophase) is probably the best parameter of centralization, even in these non-sinusoidal cases.

Before continuing with the discussion of phase indicators, it should be noted that, as in the case of scalar magnitudes, it is advisable to use non-overlapping sections with s = l = T (daily sections), since we are analyzing a value (a phase) whose definition involves a complete cycle (only one T) and also assumes that the value can change from day to day.

To avoid the problem of lack of fit between the data and the sine function, in most studies the center of gravity of data is used [16-18]. Conceptually, this procedure does not prejudge the profile of the series and shows, in a totally reliable way, the “temporal center” of the variable studied. The center of gravity in a time series was defined by Kenagy [19] as the mean time of the activity events. To calculate the phase corresponding to the center of gravity in an evenly sampled series, we can use the formula:

$$\phi_{\mathrm{cog}}=\frac{2\pi }{n}\frac{\sum_{i=1}^{n}iy_{i}}{\sum_{i=1}^{n}y_{i}}$$

Despite the advantages already mentioned, this method has practical drawbacks to maintain the wave profile more or less centered in the range of the analyzed section.

**Figure 5:**
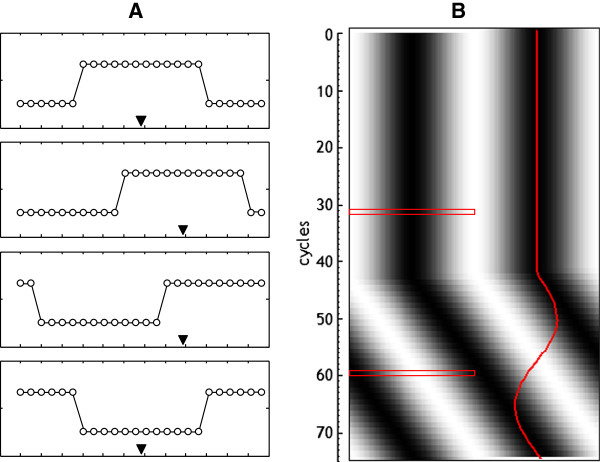
**Calculation of center of gravity. A**: effect of the displacement of the wave with respect to the section in the calculation of the c.o.g. (black triangles). **B**: serial calculation of the c.o.g. (red line) in a simulated sinusoidal wave, with s = l = T. Red rectangles show two sections to illustrate the displacement of the wave with respect to the section. If this displacement is not compensated, the erroneous estimation is clearly visible on the resulting graph.

Suppose the case of a perfect square wave. In this case, if the start of the wave (positive edge) moves, the center of gravity of the wave will move in the same manner, since the entire wave falls within the range tested. But if the displacement of the wave is such that the wave starts at a point in the cycle, but the end is located beyond the range tested, then the final portion of the wave moves to the beginning of the cycle, and the center of gravity will be located erroneously in the lower part of the wave, as shown in Figure 5A. To avoid this error the analyzed section must be redefined each cycle. However, in a real series, the difference between high and low values is not as marked as in the example, which makes it virtually impossible to make such adjustments. Figure 5B shows how the center of gravity would change in a simulated sinusoidal series.

The solution to the problem of having to modify the section analyzed in each cycle (it requires an approximate knowledge “a priori” of the phase changes) is to calculate the center of gravity in a circular manner, rather than linear in time. This assumes that the different values of the series are distributed in a unit radius circle and each point represents a mass equal to its value. Thus the coordinates (a, b) of the center of gravity is calculated by the formulae:

$$a=\frac{\sum_{i=1}^{n}y_{i}\cos\frac{2\mathrm{\pi i}}{n}}{\sum_{i=1}^{n}y_{i}};\;b=\frac{\sum_{i=1}^{n}y_{i}\sin\frac{2\mathrm{\pi i}}{n}}{\sum_{i=1}^{n}y_{i}}$$

**Figure 6:**
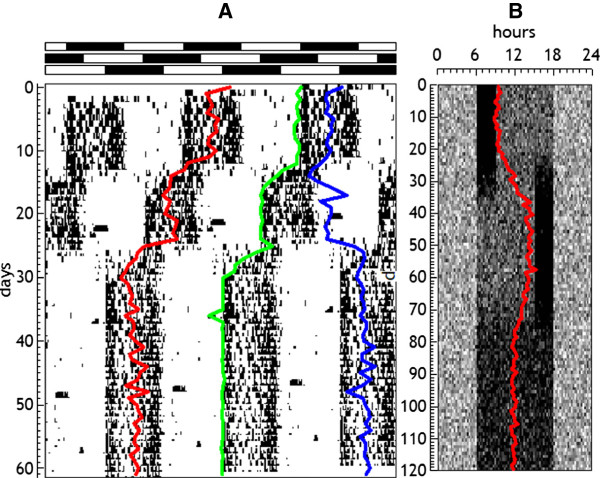
**Estimation of acrophase. A**: evolution of acrophase (red), positive flank using Heaviside function (green), and center of gravity (blue), in a real series of motor activity from a mice submitted to 8 h advances in the light–dark cycle. **B**: evolution of the acrophase in a simulated series with one bout of activity at the beginning and the end of a square wave, to show the effect of the wave shape on the estimation of the acrophase. In both cases de sections are s = l = T.

The angular position of the center of gravity (a, b) is the phase, and its tangent is the ratio b/a, so that if we calculate this ratio, we obtain:

$$\frac{b}{a}=\frac{\sum_{i=1}^{n}y_{i}\sin\frac{2\mathrm{\pi i}}{n}}{\sum_{i=1}^{n}y_{i}\cos\frac{2\mathrm{\pi i}}{n}}$$

since the terms $\sum_{i=1}^{n}y_{i}$ cancel out. This expression is the same as the formula for calculating the tangent of acrophase, so that the acrophase exactly equals the center of gravity, independently of the position of the beginning of the analyzed section. This is a very important result and is for that reason (acrophase coincides with the circular center of gravity) that the acrophase can be considered the best parameter of centrality. Figure 6A shows the evolution of acrophase in a real series, jointly with other parameters to be discussed below, and Figure 6B shows how the estimation of the acrophase is affected by changes in the shape of the rhythmic pattern.

Regardless of the parameter of centrality, in numerous studies the focus is on determining the start of the active phase or the end of it, especially when patterns have non-sinusoidal waveforms (generally square) or when working under the hypothesis of various oscillators (e.g. morning and evening components [20]). It is also common to study the two points simultaneously, and such is the case of studying the duration of the alpha phase [9]. Again, if the contrast between the phases of activity and rest is marked, the estimation of these parameters is relatively simple. Often this estimate is done “*de visu*” on the graph itself (“double plot”), where the values are estimated graphically, but it is possible to use analytical methods that are more accurate and totally objective, such as those shown below. We must bear in mind that if the studied variable X has a certain inertia (e.g., temperature) the use of graphical methods can be very complicated, because of the difficulty of establishing the threshold between high and low values.

The procedure that often works best is to perform (for each section) a regression analysis between the data and the Heaviside function (the unit step function) using a variable displacement ψ between the series and the Heaviside function. The offset value ψ varies from 0 to n, and the value of ψ at which the higher correlation index is obtained is chosen as the indicator of the start of activity phase. Mathematically, Heaviside function is represented by the letter H, but to avoid confusion with the transfer function used above, we’ll use the symbol θ, which is also used in some cases of continuous functions. This function is defined as θ(t) = {0: t < c, 1: t ≥ c}, i.e. is zero for any time lower than an arbitrary value c, and 1 for times greater than or equal to c, thus representing a unit step. The procedure, in fact, looks for the time point when the data set has a behavior similar to θ(t). Formally at that point there is a “positive flank”, which is indicated by the symbol ψ+. To calculate the end of the phase of activity, one uses the same procedure but using the complementary function defined as θ'(t) = 1-θ(t) = {1: t < c, 0: t ≥ c }, defining a negative step, so that the point found corresponds to the negative flank ψ-.

**Figure 7:**
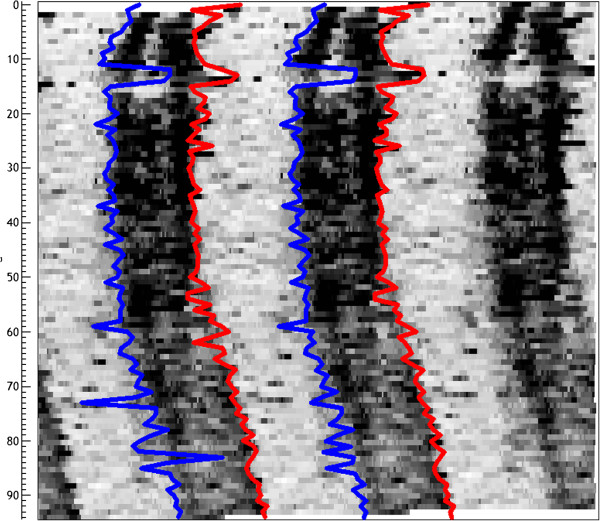
**Onsets and offsets.** The figure is a triple plotted actogram from a rat, with the positive flanks (blue) and the negative flanks (red) calculated by fitting dichotomized data to a squared wave (left) and to a Heaviside function (right). Small differences are visible in the two methods.

There are variants to improve the calculation for the estimation of the flanks, consisting in performing the regression analysis with a modified set of data instead of with the original data series. The transformation consists in “dichotomizing” the series by a threshold u, so that the transformed series is defined as y' = {0: y < u, 1: y ≥ u}. Thus we obtain a series consisting of zeros, when y is below u and ones when above. The threshold u is usually defined by the mean or (better) by the median of Y. It is also possible to use a percentile value, depending on the distribution of values on the series Y. The choice of threshold requires some knowledge of the characteristics of the series and is frequently accomplished by tests and trials, comparing the graphs of the ψ values obtained on the dataset itself. Figure 7 shows the curves of negative and positive edges over the actogram of a real series.

Another possibility to estimate the position of flanks is to use a square wave for the regression instead of the Heaviside functions (see Figure 7). This procedure improves slightly the previous one because the Heaviside function only takes into account an increase (or decrease) on the series values, which means that a part of the series is not properly adjusted to the actual values because in the real data set both an increase and a decrease take place. To solve this problem a first estimate of the positive and negative flanks is made using the above procedure. After that, the positive flank is kept (c = ψ+) and successive regression analysis are performed, as in the previous case, but with a square wave function q(t) = {1: c ≤ t < d, 0: otherwise} and changing d till finding the best fit that will define the negative flank d. Then the procedure is repeated but keeping d, and obtaining a new value for c. These new values of flanks, c and d, are compared with the values ψ + and ψ- obtained initially. If they are equal, they are considered valid, but if different, the procedure is repeated using the new values c and d as the initial estimates of flanks, and the procedure continues until the two pairs of values coincide. Although the procedure is a bit complex, it can be automated, and has the advantage that it gives very good estimates, especially in series with irregular circadian patterns.

Finally, we will discuss other methods for estimating parameters of centrality or flanks, which are much simpler, but their application is restricted to highly uniform and noise-free series. It consists simply in determining the time at which the series has its minimum value or its maximum value, or the point at which the greater increase or decrease takes place between two successive values (positive and negative flanks respectively). To apply these methods, a previous smoothing of considerable amplitude is necessary. Another possibility is locating the point at which the data series crosses a certain threshold (mean, median or percentile), but here it is necessary to apply a moving average with an amplitude close to one third the length of the cycle analyzed, to remove local variations. A serious inconvenient of these techniques is that the smoothing effect can be so great that they cause distortion in the wave and errors in the estimation of parameters. Therefore these techniques are not very suitable and should be used only in the case of very “soft” waveforms.

A consideration which is common to all angular magnitudes that have been considered in this section is the monitoring of the phase changes between sections, to avoid changes greater than half a cycle T/2 = π rad = 180°. In an example where the sections are analyzed with T = 24 hours, it could be possible, as a result of a calculation, to obtain a phase shift of 18 hours, which actually corresponds to a change of −6 hours, considering the shorter jump. In this case, one would have to subtract 24 hours (T = 2π rad = 360°) from the calculated value to obtain the correct value. It must be noted that the value of the phase shift has to be calculated from the expected value in the corresponding section and not the absolute value of the phase. Suppose we obtain values 4 h, 6 h and 8 h in subsequent sections. If in the next section we obtain 10 h, it is clear that it is equal to the expected value and the phase shift from the expected value will be zero, although there is an absolute increase of 2 h, with respect to the previous section. To monitor the phase and make corrections properly, one must take the k values of the preceding sections and extrapolate the next point on the basis of linear regression with the previous points. It is possible to obtain relatively simple formulae for this extrapolation for fixed values of k ≤ 6. In this way, false jumps will be eliminated in the calculated series of phases.

#### Magnitudes related to frequencies (or periods)

The main property of a rhythm is its period (or frequency, which is the inverse of the period). After knowing the period, the amplitude of the oscillation is the characteristic that best indicates the importance or the magnitude of the rhythm. In addition to quantifying the amplitude, one can use other related measures to determine the importance of a rhythm, such as the percentage of power or variance. In principle, the simplest oscillation and the one than least prejudges the pattern of a rhythm corresponds to a sinusoidal oscillation. For this reason the first analysis tool will be the fitting of data to a sinusoidal function, which is known as “periodic regression” [21]. This technique, classical in circular statistics, estimates the amplitude and the acrophase of the rhythm present in a data series [22]. This technique has been adapted for the analysis of circadian rhythms using a characteristic polar representation that facilitates the visualization and analysis of the confidence intervals of the estimated parameters, leading to the “cosinor” method [23]. When the pattern is more complex than a simple sinusoidal function, the adjustment can be made to a function that includes a number of sinusoidal components, each with a period that is sub-multiple of the main period T:

$$\begin{array}{c} y\left(t\right)=c_{0}+c_{1}\cos\left(\mathrm{\omega t}-\theta_{1}\right)+c_{2}\cos\left(2\mathrm{\omega t}-\theta_{2}\right)\ldots \\ +c_{h}\cos\left(\mathrm{h\omega t}-\theta_{h}\right);\;\omega =\frac{2\pi }{T} \end{array}$$

$$y\left(t\right)=c_{0}+\overset{h}{\underset{i=1}{\sum }}c_{i}\cos\left(\omega_{i}t-\theta_{i}\right);\;\omega_{i}=\frac{i2\pi }{T}\;$$

where c_i_ and θ_i_ are the amplitude and phase of each component i. Each one of these components is called a harmonic, and the graphical representation of the amplitudes of the successive harmonics is the spectrum of the series. This spectrum is characteristic for each rhythmic pattern shape and clearly indicates the different frequency components involved in defining a pattern. The decomposition of a waveform into its harmonic components is done by spectral analysis or Fourier analysis [24,25].

This type of analysis can also be done serially on sections that may overlap or not. In case of overlapping sections, as already mentioned in previous cases, the procedure will produce a smoothing over the values of all the parameters resulting from the analysis. If one makes a serial Fourier analysis, it is important that the length of the analyzed sections is equal to or an integer multiple of the period used for the main analysis. This is because under this condition (orthogonality), the estimates of harmonics are independent of the other components, so that if one harmonic has certain amplitude, this does not affect the magnitude of another harmonic, while if the condition of orthogonality is not satisfied, the estimation of each harmonic affects the other components, which leads to an erroneous or biased spectrum. In this type of analysis, it is particularly useful to employ power spectrum (instead of the amplitude) in which the magnitude of each harmonic is expressed as the fraction that represents the square of its amplitude with respect the sum of the squares of all harmonics. This allows to quantify the importance of each component as a rate (or percentage), regardless of its real amplitude. In this case the spectrum is called power spectrum.

In all these analyses, one must establish a value for the period, that must be known or estimated “a priori”. This is the fundamental period to be used throughout the whole analysis and that, logically, will be the rhythm of the series. When this period is not known with accuracy, we shall see other techniques aimed precisely at determining the value of the period of the rhythm.

**Figure 8:**
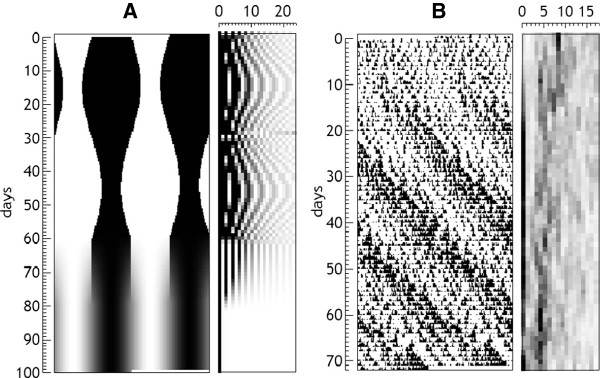
**Graphic representation of the evolution of power spectra for a synthetic and a real series.** In both examples double plotted actogram is at the left of the graphic matrix in which each column corresponds to a harmonic. In **A**, a simulated series with a rectangular pattern that changes in length becoming symmetric and then changing to a sinusoidal. The characteristic spectrum of rectangular waves is clearly visible. When symmetric, even harmonics are null, and all the harmonics disappear but the 1st, when the sinusoidal pattern appears. In **B**, a real record of a young rat after weaning under constant light, clearly showing the transition of an ultradian pattern characteristic of an immature animal (harmonics around 8), to the characteristic circadian pattern of an adult (power in the 1st harmonic).

In the case of the serial analysis of these magnitudes, the most frequent is to follow the evolution of the amplitude (or power) of the first harmonic. It is also possible to follow the evolution of several components at once, but this results in very complex graphs in which the lines for each harmonic intersect and it is almost impossible to visualize the evolution of each one of the curves. In some cases it may be of interest to study the power of several harmonics at a time (typically, the sum of the first components). In any case, there is a graphic method for the representation of the successive spectral analysis that perfectly shows the evolution of the different components over time. This representation consists of making a graphic matrix [26] in which each row corresponds to the analysis of one section (usually one day or one cycle) and each column to a harmonic. Each cell of the matrix is colored with a color (or gray intensity) proportional to the power of the harmonic corresponding to the row (section) analyzed. To improve visualization of changes in each harmonic, a vertical moving average may be applied, as shown in Figure 8. This figure shows the corresponding graphical matrices for a real series and a synthetic series gradually passing from a square wave pattern to a sinusoidal. This example clearly shows the evolution of the different components that make up the square wave. Importantly, the presence of harmonic components in the spectrum does not necessarily indicate the presence of rhythms other than the main one, but often they are constitutive components which are integrated in the definition of a specific pattern and should not be interpreted as expressing oscillations other than the fundamental period. In the case that the amplitude of a harmonic component is greater than the main component, then it would be justified to presume the presence of a differentiated basic rhythm. We have already mentioned that the spectra are characteristic for each rhythmic pattern, or waveform. Thus, in Figure 8A, it is clear that in the spectrum corresponding to the square wave, even harmonics are zero, which is a characteristic of symmetrical waveforms, and the amplitude modulation of the sinusoidal component is a characteristic of rectangular shapes. With some experience it is possible to deduce some features of the waveform from the inspection of the spectra. In the case of Figure 8B, one can see the transition of an ultradian pattern characteristic of an immature animal to the characteristic circadian pattern of an adult.

**Figure 9:**
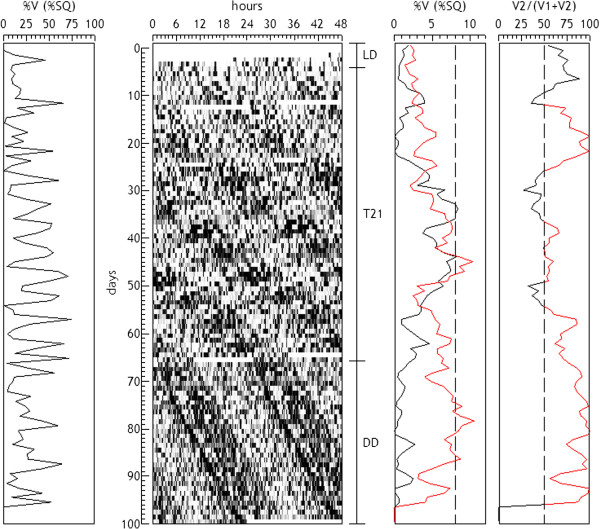
**Serial analysis of the motor activity of a rat maintained under LD cycles with a period of 21 hours.** The graph shows the simultaneous presence of the animal's own endogenous component (T2 = 1540) and the component entrained by the light (T1 = 1260). Sections are s = 1440 min and l = 7200 min. The analysis consisted in a linear model including simultaneously the two sinusoidal components (T1 and T2). In the graphs are represented: the total variance explained by the model (left), the variance explained by each component (3rd graph, black = T1, red = T2) and the percentage of variance explained by T2 with respect to the sum of two components (right).

A special case occurs when a series presents two simultaneous rhythms with close periods [27]. In this case it is not possible to define a section length containing an integer number of cycles for the two components, so that the orthogonallity condition is not satisfied, and consequently the parameters of the two rhythms cannot be estimated independently. In these cases one must chose sections containing at least 2 or 3 full cycles and make an adjustment using a linear model that includes the two periods, which must be known in advance. Thus it is possible to calculate the ratio of the amplitudes (or power) between the two components, although the absolute individual estimates are always modified by the presence of the other component (non-orthogonal). Figure 9 shows an example of this type of analysis, corresponding to the motor activity of a rat maintained under LD cycles with a period of 21 hours, which shows the simultaneous presence of the animal’s own endogenous component and the component entrained by the light.

#### Periodograms

In the previous section, quantities related to specific frequencies (or periods) were calculated, meaning that the value of the frequency at which the data should be adjusted is known or assumed in advance. Now we will deal with the case in which the value of the frequency (or period) is unknown, and the aim of the analysis is precisely finding its value. For these calculations a spectrogram can be used, or one may conduct a serial analysis consisting of successive periodograms. Usually, quite long sections are required, which must encompass a minimum of 8–10 days (or cycles). The jump between successive sections usually is a complete cycle. Thus what is obtained is a daily succession of periodograms. Each periodogram is a row in a graphic matrix and each cell will correspond to one of the tested periods in the specific section, colored according a gray scale, or other chromatic scale. The most suitable periodograms for this kind of representations are the Lomb-Scargle [28,29] and the Sokolove-Bushell [30]. The first one should be used when sections are shorter than 8 cycles.

Each periodogram has its own characteristics, whose discussion is beyond the scope of this article. Everything that can be explained in the “normal” periodograms (not serial) also applies in this serial application. Before explaining the differences and characteristics of these two periodograms we should insist on a common feature of all periodograms, for a proper interpretation. This is the fact that the values shown in a periodogram are obtained independently of each other, and not in a jointly way. This means that if in three adjacent points of a periodogram corresponding (for example) to periods of 1200, 1210 and 1220 minutes have values of 35, 38 and 42% of the total variance, that does not mean that each of these periodicities are present on the wave with such percentages of variance simultaneously. The true interpretation is that when a period of 1200 minutes is tested, this period explains 35% of the total variance, and if we perform a new (and different) analysis with a period of 1210 minutes, then it explains 38%. Therefore, each tested period is an independent test, performed over the same dataset, which obliges to apply the Bonferroni correction (explained above) on the probability level required in each test (or point) to maintain the desired global level of significance.

It should be noted that in the case of serial Fourier analysis (graphic matrices) several frequencies are also shown simultaneously for each section, but in that case the different harmonics are calculated simultaneously and orthogonally, so that the percentages of variance correspond to the distribution of the total variance of series, among the different harmonic components, while in the periodogram this is not so. Although the two periodograms generate a Chi2 type variable, one can transform these values in percentages of explained variance, which is much easier to interpret.

The first periodogram to consider will be the periodogram of Sokolove-Bushell (SBP). Its main feature is a high sensitivity to the repeatability, cycle by cycle, of any rhythmic pattern, even if far from sinusoidal. The formula used for the calculation of Q_P_ is

$$Q_{p}=\frac{\mathrm{KN}\sum_{h=1}^{P}{\left({\bar{y}}_{h}-\bar{y}\right)}^{2}}{\sum_{j=1}^{N}{\left(y_{j}-\bar{y}\right)}^{2}}$$

where P is the period expressed in samples, ${\bar{y}}_{h}$ are the column means after arranging the series (of N elements) in an array of P columns, and K is the number of rows of the resulting array. Q_P_ follows a Chi^2^ distribution with as many degrees of freedom as cycles in each section (see a description of the method of calculation in [30]). From the value of Q_P_, the amount of variance explained by the rhythm can be calculated [31] just multiplying Q_P_ by 100/N.

The Lomb-Scargle periodogram (LSP) has been proposed in the field of Chronobiology more recently and has some outstanding features: It can be applied to series with non-uniform sampling, is very sensitive to the presence of any rhythmicity and is not affected by the subharmonic components of the principal one. This means that if there is a rhythm of 500 min in the series, logically, there will also be periodicities with T equal to: 2 · 500 = 1000, 3 · 500 = 1500, 4 · 500 = 2000 minutes, etc. In the SBP these periodicities would appear in the graph, while in the LSP, they will not be present, and only the 500 minutes component will clearly be shown. There is abundant literature [32,33] where you can find the details of the methodology used. The following formulae are used to compute the LSP, P(ω):

$$P\left(\omega \right)=\frac{1}{F}\left(\frac{\mathrm{CY\cdot CY}}{\mathrm{CC}}+\frac{\mathrm{SY\cdot SY}}{\mathrm{SS}}\right);\;\omega =\frac{2\pi }{T}$$

with T expressed in samples, F is two times the variance of y, and the sums are (for simplicity, in the next formulas, $\sum_{i=1}^{N}\cdots \equiv \sum \cdots )$:

$$\mathrm{CY}=\sum \left(y_{i}-\bar{y}\right)\;\cos\;\left(\omega \left(t_{i}-\tau \right)\right)$$

$$\mathrm{SY}=\sum \left(y_{i}-\bar{y}\right)\;\sin\left(\omega \left(t_{i}-\tau \right)\right)$$

$$\mathrm{CC}=\sum \mathrm{co}s^{2}\left(\omega \left(t_{i}-\tau \right)\right)$$

$$\mathrm{SS}=\sum \mathrm{si}n^{2}\left(\omega \left(t_{i}-\tau \right)\right)$$

the correction term τ is calculated for each ω:

$$\tau =\frac{1}{2\omega }\arctan\frac{\sum \sin2\omega t_{i}}{\sum \cos2\omega t_{i}}$$

The maximum value of P(ω) is (N-1)/2, and the threshold for a level of significance equal to p (including the Bonferroni correction) is

$$P_{p}=\frac{N-2}{2}{\left(1-\left(1-p\right)\frac{1}{N}\right)}^{\frac{2}{N-3}}$$

When using relatively short sections, there is a slight underestimation of the long periods. To avoid this we can use the “generalized floating mean” LSP [34] using the same formulae but with several changes: before the calculations , the values y_i_ must be normalized to N(m = 0,s = 1), and

$$\tau =\frac{1}{2\omega }\arctan\frac{\sum \sin2\omega t_{i}-2\sum \mathrm{sin\omega}t_{i}\sum \mathrm{cos\omega}t_{i}}{\sum \cos2\omega t_{i}-\sum {}^{2}\mathrm{sin\omega}t_{i}+\sum {}^{2}\mathrm{cos\omega}t_{i}}$$

$$\mathrm{CY}=\sum y_{i}\mathrm{cos\omega}\left(t_{i}-\tau \right)$$

$$\mathrm{SY}=\sum y_{i}\mathrm{sin\omega}\left(t_{i}-\tau \right)\cdots$$

$$P_{p}={\left(1-\left(1-p\right)\frac{1}{N}\right)}^{\frac{2}{N-3}}$$

All other formulas remain the same as in the previous periodogram. The maximum value of P(ω) is 1, when the periodicity accounts for all the variance of the series; because of this, 100· P(ω) can be interpreted as the percentage of explained variance.

**Figure 10:**
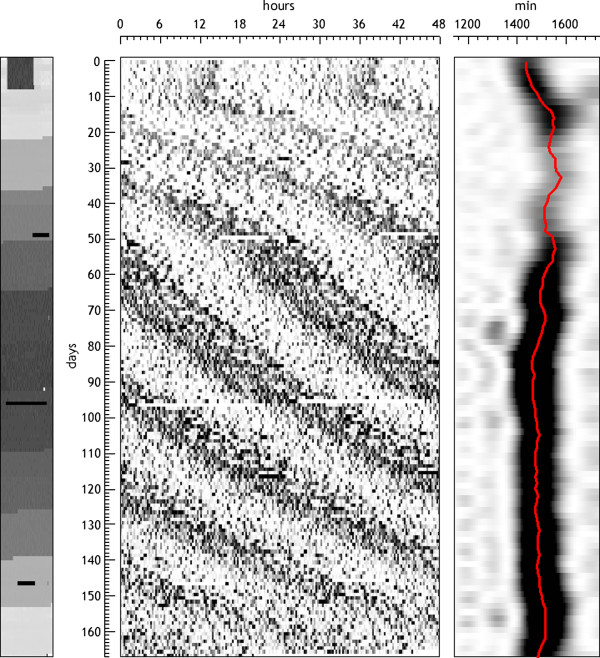
**Motor activity of a rat submitted to different constant light intensities.** The serial periodogram is shown on the right; the different light intensities are shown on the left. The periodogram used was the Lomb-Scargle with a section of 10 days length and a step of 1 day, and it is represented in a gray scale. The red overpainted line corresponds to the daily estimated period (maximum of the periodogram).

In both methods the periodograms can be normalized to have the maximum equal to a constant. This facilitates the identification of the highest peak in each section, but has the drawback that it does not reveal the changes in the degree of presence of rhythmicity along all the serial analysis. Another possibility that is derived from this type of analysis is to represent, for each section, the period value at which the maximum occurs, thus allowing one to follow the evolution of the main period over time (Figure 10).

**Figure 11:**
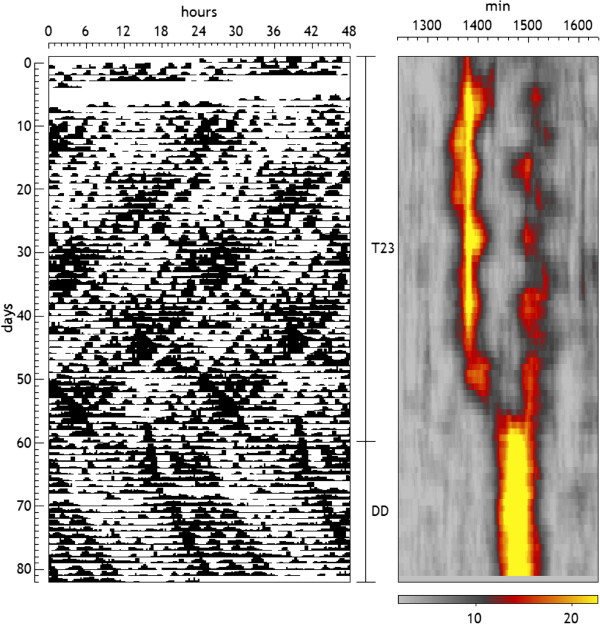
**Motor activity record of a rat submitted successively to LD: T = 23 and DD.** The serial periodogram is shown on the right. The periodogram used was the Sokolove-Bushell with a section of 20 days length and a step of 1 day, and the percentage of explained variance is represented in a color scale shown below. The presence of two components is clearly visualized during T23, changing to a unique rhythm in DD with an intermediate period.

In the case that several frequencies are expressed, this technique allows to clearly visualize its evolution, as shown in Figure 11, in which, again, individual peaks don’t represent the degree of simultaneous presence of frequency components but its significance when analyzed separately in the series. To study the degree of simultaneous presence of two components of known frequencies, one should apply the procedure described at the end of the previous section.

#### Derived and/or special magnitudes and variables

The number of variables that can be studied in a serial analysis is virtually unlimited, since it is possible to define a multitude of indices and variables from those already indicated or others different, depending on the properties under investigation. As example, a few cases will be discussed.

One case is the duration of the activity phase or alpha phase. It can be expressed in absolute terms or as a percentage with respect the duration of the cycle. To calculate the length of the alpha phase, one can calculate the difference between the positive and the negative flank, as already calculated above.

Some authors determine the degree of entrainment, expressing the portion of activity that takes place during a certain part of the light cycle [35]. To calculate this value, one uses sections with a length and step equal to the period of the cycle of illumination, and then one calculates the amount of activity in the first half of the section, with respect to the activity recorded in the entire section.

Other specialized variables for actigraphic data can be calculated and represented in a sequential manner like those proposed by Van Someren et. al. [36] to study the activity-rest cycles. The IV (intradaily variability) assesses the fragmentation of the rhythm, based on the frequency and extent of transitions between active and inactive hours. Assuming a sampling interval of 1 h, the IV variable is calculated by the formula:

$$\mathrm{IV}=\frac{N}{N-1}\frac{\sum_{i=2}^{N}{\left(y_{i}-y_{i-1}\right)}^{2}}{\sum_{i=1}^{N}{\left(y_{i}-\bar{y}\right)}^{2}}$$

This index has proved to be useful to follow the effect of treatments in demented patients in long periods of time, and because of this, it is well suited for its serial application.

The RA (relative amplitude) is the other proposed index, as a non-parametric alternative to the estimation of the sinusoidal amplitude. RA is calculated by the formula:

$$\mathrm{RA}=\frac{M_{10}-L_{5}}{M_{10}+L_{5}}$$

Where M_10_ and L_5_ are the activity values for the most active 10 hours period and the least active 5 hours period in the average 24 hours pattern (sections should include 5 or more cycles). This index provides a good indication about the degree of coupling between the activity rest cycle and the external *zeitgeber* (light–dark cycle).

### Wavelet analysis

The wavelet analysis is a relatively new technique for analyzing a process in time and frequency simultaneously. Its main advantage is that the process does not require the date to be stationary or to have a constant spectral structure, so it is especially suitable for the analysis of rhythmic processes whose characteristics vary in time. Although there is not a serial analysis technique, the calculation methodology has many similarities to this type of analysis, so here we will make brief reference to these techniques and refer the reader interested in them to the extensive mathematical literature existing and more specifically to two recent articles [36,37] on its application in chronobiology.

Its application in chronobiology is scarce and there is no clear consensus on how to implement this technique. In addition to the articles mentioned, early studies were conducted in the late 1990s in which wavelet analysis was used for the characterization of ultradian rhythms [38], for monitoring phase changes [39] or for signal recognition [40] and more recently in studies on variations of the period [41].

The analysis is performed by dividing the series in different sets of sections with lengths that are half of the previous one, and their associated frequencies will double. In each resulting set of sections a wavelet with the same length of the section is applied to the data by cross-multiplying their terms. Wavelets are shrunk or lengthened to fit the section length. They are not sinusoids and have very characteristic waveforms that form a set of orthogonal functions with a domain of existence limited by the length of each section (this interval defines the “support” of the wavelet). This type of analysis has a clear discrete character (DWT Discrete Wavelet Transform), so it could be considered similar to performing various serial analyses with non-overlapping sections with a different length and step (each length is associated to a frequency) in each analysis.

**Figure 12:**
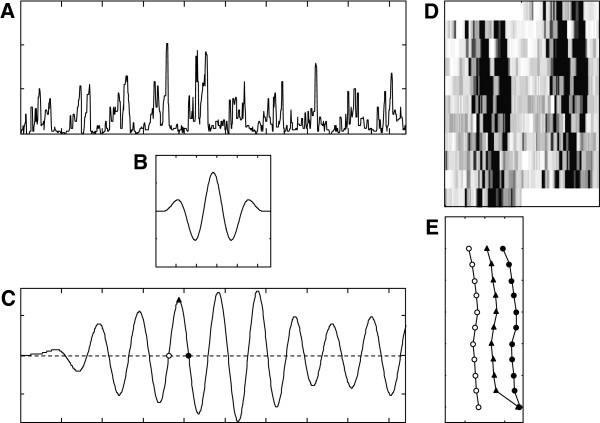
**Wavelet analysis: procedure.** The original series **A**, from a real motor activity record **D** is convolved with a sinusoidal-Gaussian-modulated function **B** obtaining the curve C after numerical smoothing. For each resulting cycle in **C**, three characteristic points are defined: when crossing zero upwards (the onset), the maximum (the middle) and when crossing zero downwards (the offset). In **E**, the evolution of these points cycle by cycle.

**Figure 13:**
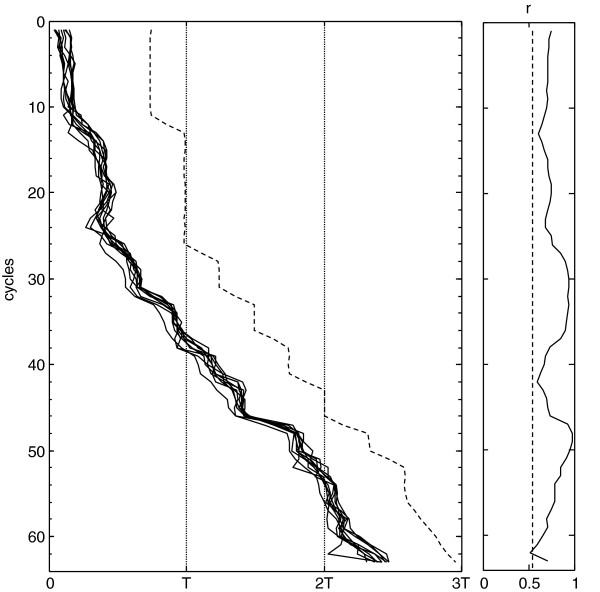
**Wavelet analysis: result.** Shown is the representation of the evolution of the phases (continuous lines) calculated with the convolution described in Figure 12, on 10 registers of motor activity of rats subjected to continuous phase changes of lighting cycle (dashed line). At right, the result of the Rayleigh z test, to verify the homogeneity of the daily phases, dashed line is the threshold for significance (p = 0.05).

Another type of the wavelet analysis that will be discussed here is primarily used to locate markers of the rhythm phase (start of activity, central point, etc.). This variant is the continuous analysis (CWT, Continuous Wavelet Transform) which is just the convolution of a specific wavelet over the data series [42]. It uses a collection of identical wavelets but with lengths (periods) continuously decreasing. So, one obtains a continuous series of convolutions for the different periods studied. From all possible periods, the one that corresponds to the period of the process studied is selected. Regarding the choice of the wavelet, there is a wide variety of them, and the selection of the most suitable type depends on several factors, including the similarity with the studied wave pattern, which would be the one with most relevance. It is also possible to define a wavelet specially “tailored” to the data. If the wave pattern is not known, it may be defined as a sine wave modulated by a Gaussian function such as shown in Figure 12B. Using a function of this type, with a support for 3 full cycles, one can obtain a convolution g(t) from which the phase indicators can be detected. To do so, a smoothing function must be applied to the resulting convolution g(t) to remove the effect of noise and then a threshold must be set to detect the beginnings of activity, when g(t) crosses the threshold upward. The indicator of the central point of each cycle corresponds to the maximum of g(t), as shown in Figure 12C. In Figure 13, we have analyzed 10 registers of motor activity of rats subjected to continuous phase changes of the lighting cycle, using the technique described. The graph shows the good tracking of the individual phases, and also (at right side) the result of the Rayleigh z test [22], to verify the homogeneity of the daily phases in the group of rats studied. This test estimates the module (r) of the average vector of the unit vectors corresponding to the phases of each individual using the formula:

$$r=\sqrt{{\bar{a}}^{2}+{\bar{b}}^{2}}$$

$$a_{i}=\cos\theta_{i},\;b_{i}=\sin\theta_{i}$$

where a_i_ and b_i_ are the orthogonal unit components of each phase angle θ_i_. The higher the value of r the greater degree of homogeneity in phases. The significance threshold of r, for a probability p = 0.05, can be calculated by the formula:

$$r_{\left(0.05,v\right)}=1.6732268/v^{0.492018,}$$

where v is the number of cases.

### Practical application and conclusions

The serial analysis of long duration data series includes a wide variety of methods, such as we have seen, and it is not easy to find applications capable of performing these calculations in a compact form. Consequently, one often needs to develop specific programs or routines to perform the calculations.

There is a specific application for chronobiology that can perform all the methods described in this article, with the exception of wavelets. This is the application “El Temps” by Díez-Noguera [http://www.el-temps.com], with which most graphics of this article were produced. Other programs which may be used as the basis for such calculations are some classical ones for the analysis of signals, such as TISEAN by Hegger, Kantz & Schreiber [http://www.mpipks-dresden.mpg.de/~tisean/] which includes spectral analysis, periodograms, and a powerful set of tools for nonlinear analysis. There are also a number of specific applications for chronobiology and biological rhythms analysis which, in general, focus on the adjustment to sinusoidal functions, cosinor, periodograms, actograms and waveform analysis. Although these applications are not designed specifically for serial analysis, they may be very useful to perform the analysis of successive sections manually. Below is a list of programs (in alphabetical order) that can be used for this purpose:

“ActogramJ” Java app by Schmid, Helfrich-Föster & Yoshii [http://actogramj.neurofly.de],

“BRASS” Excel app by Millar [http://millar.bio.ed.ac.uk],

“Chronos-Fit” by Lemmer [http://www.fileguru.com/Chronos-Fit/info],

“Circadian software” by Refinetti [http://www.circadian.org/main.html],

“ClockLab” from Actimetrics [http://www.actimetrics.com/ClockLab/],

“CronoLab” by Mojón, Fernández & Hermida [http://www.tsc.uvigo.es/BIO/Bioing/ChrLDoc1.html],

“El Temps” by Diez-Noguera [http://www.el-temps.com],

“Free chronobiology software” by Hut [http://hutlab.nl],

“The Chronobiology Kit” from Stanford Software Systems [http://query.com/chronokit/],

“Time Series Analysis-Cosinor” from Expert Soft Technologie [http://www.euroestech.net/index.php].

In the case one prefers to write his/her own programs, one can use any of the many existing programming languages -- such as Delphi, Basic, C++, C#, and Java --, several of which include programming environments that facilitate the work very much. A serious disadvantage is the requirement of specific programming skills, which most users do not possess. For this purpose there are mathematical applications that use a special language for commands, operations and functions, such as MATLAB, allowing the execution of small programs or scripts that are extremely useful for such calculations. This language includes a great number of predefined functions (statistics, graphs, spectral analysis, regression, etc.) that facilitate the realization of the calculations automatically. Because it is a very high level language, many processes are preprogrammed and ready to use, which saves programming time and effort. It is worth to point out that MATLAB is a language that can be handled relatively easily, at very acceptable levels without high expertise, and this explains its great popularity and acceptance in the field of computing and numerical analysis. The existing large user community has developed many applications that are freely available in the Internet, and in many cases it is not difficult to find a program that suits one’s specific needs.

Another similar application is Mathematica, with similar characteristics as MATLAB but with different programming strategies. Both programs have additional libraries that extend the capabilities of analysis to specific fields, such as statistics, waveform analysis, wavelet analysis, filters design, etc. all of them very useful for our purpose of analysis.

In short we can say that the serial analysis is a tool of the first order to analyze the rhythmic characteristics of long time series, in which rhythm properties evolve over time. In fact it is nothing else that the repeated application of conventional rhythm analysis techniques along different sections of a time series. As already mentioned in this article, the correct application of these methods requires some considerations and precautions, already mentioned above, to avoid the commission of errors, as when choosing the length of sections and the step size. Likewise the interpretation of the results must be conducted cautiously under the knowledge of the specific characteristics of each technique, as in the case of Fourier or spectral analysis.

In general terms, one can say that when a time series includes more than 20 successive cycles, it is necessary to use the serial analysis to see the evolution and changes that have taken place during that time period. If this analysis is not performed and the whole data series is treated as a single unit, the presence of errors is highly likely due to the non-stationarity of the process, since most of the conventional analysis methods are defined for stationary series whose properties remain constant throughout the investigated period, a situation that is very rare in living beings.

Although most of the techniques described here have a rather complex mathematical basis, the existence and availability of numerous computer applications, more or less specific, greatly facilitates the practical implementation of these methods. We believe that the systematic application of these analytical techniques, would improve the description of many experimental observations allowing a more accurate analysis.

## Competing interests

The author declares that he has no competing interests. The “El Temps” software is a commercial product produced by Antoni Díez-Noguera.
